# Goats naturally devoid of PrP^C^ are resistant to scrapie

**DOI:** 10.1186/s13567-019-0731-2

**Published:** 2020-01-10

**Authors:** Øyvind Salvesen, Arild Espenes, Malin R. Reiten, Tram T. Vuong, Giulia Malachin, Linh Tran, Olivier Andréoletti, Ingrid Olsaker, Sylvie L. Benestad, Michael A. Tranulis, Cecilie Ersdal

**Affiliations:** 1 Department of Production Animal Clinical Sciences, Faculty of Veterinary Medicine, Norwegian University of Life Sciences, Sandnes, Norway; 2 Department of Basic Sciences and Aquatic Medicine, Faculty of Veterinary Medicine, Norwegian University of Life Sciences, Oslo, Norway; 3 Norwegian Veterinary Institute, Oslo, Norway; 4 UMR1225, INRA-ENVT, École Nationale Vétérinaire, Toulouse, France

## Abstract

Prion diseases are progressive and fatal, neurodegenerative disorders described in humans and animals. According to the “protein-only” hypothesis, the normal host-encoded prion protein (PrP^C^) is converted into a pathological and infectious form (PrP^Sc^) in these diseases. Transgenic knockout models have shown that PrP^C^ is a prerequisite for the development of prion disease. In Norwegian dairy goats, a mutation (Ter) in the prion protein gene (*PRNP*) effectively blocks PrP^C^ synthesis. We inoculated 12 goats (4 *PRNP*^+/+^, 4 *PRNP*^+/Ter^, and 4 *PRNP*^Ter/Ter^) intracerebrally with goat scrapie prions. The mean incubation time until clinical signs of prion disease was 601 days post-inoculation (dpi) in *PRNP*^+/+^ goats and 773 dpi in *PRNP*^+/Ter^ goats. PrP^Sc^ and vacuolation were similarly distributed in the central nervous system (CNS) of both groups and observed in all brain regions and segments of the spinal cord. Generally, accumulation of PrP^Sc^ was limited in peripheral organs, but all *PRNP*^+/+^ goats and 1 of 4 *PRNP*^+/Ter^ goats were positive in head lymph nodes. The four *PRNP*^Ter/Ter^ goats remained healthy, without clinical signs of prion disease, and were euthanized 1260 dpi. As expected, no accumulation of PrP^Sc^ was observed in the CNS or peripheral tissues of this group, as assessed by immunohistochemistry, enzyme immunoassay, and real-time quaking-induced conversion. Our study shows for the first time that animals devoid of PrP^C^ due to a natural mutation do not propagate prions and are resistant to scrapie. Clinical onset of disease is delayed in heterozygous goats expressing about 50% of PrP^C^ levels.

## Introduction

Prion diseases are a group of fatal, neurodegenerative disorders that occur in humans and a range of animals. Among these are Creutzfeldt-Jakob disease in humans, bovine spongiform encephalopathy in cattle, chronic wasting disease in cervids, and scrapie in sheep and goats. According to the “protein-only” hypothesis, the host-encoded prion protein (PrP^C^) is converted into a pathological form (PrP^Sc^), which accumulates in the central nervous system (CNS) and variably in the peripheral tissues in these diseases [1, 2]. Studies of transgenic mice with genetic ablation of the prion protein gene (*Prnp*) have confirmed that host expression of PrP^C^ is obligatory for prion-disease development, infectivity, and neurodegeneration [3, 4]. Interestingly, the neurotoxicity of PrP^Sc^ also depends completely on host expression of PrP^C^ [5–7].

Susceptibility of small ruminants to scrapie is influenced by allelic variants (polymorphisms) in the *PRNP*. In goats, polymorphisms including S127, M142, S146, H154, Q211, and K222 have been associated with a decrease in disease susceptibility during natural outbreaks of scrapie [8–11]. Of particular interest are the S146 and K222 polymorphisms that delay clinical disease beyond the productive lifetime of goats upon experimental inoculation with scrapie [12–15]. These polymorphisms are found in a variety of goat breeds although the allele frequencies are relatively low [16–18]. In 2009 and 2017, the European Food Safety Authority (EFSA) released scientific opinions on genetic resistance in goats as requested by the European Commission [19, 20]. EFSA recommended further studies of candidate *PRNP* genotypes and allele frequencies before starting any breeding program for genetic resistance towards scrapie in goats.

In 2012, Norwegian researchers discovered a nonsense mutation at codon 32 in the *PRNP* of Norwegian dairy goats that completely terminates PrP^C^ synthesis [21]. An initial genetic survey of several unrelated Norwegian dairy-goat flocks (*n* = 192) revealed an allele frequency of the *PRNP*^Ter^-mutation of about 11% [21]. In a later genotyping of Norwegian goat bucks (*n* = 1984), 216 (10.9%) animals carried the mutation, of which only 13 (0.7%) were homozygous (*PRNP*^Ter/Ter^) [22]. Heterozygous goats (*PRNP*^+/Ter^) express approximately half the amount of PrP^C^ on the surface of peripheral blood mononuclear cells (PBMCs) [23]. This indicates that no compensatory expression from the normal allele is present.

In this proof of principal study, we investigated the predicted resistance conveyed by the *PRNP*^Ter^ allele in goats, which are the first animals described with a natural lack of PrP^C^. Groups of *PRNP*^Ter/Ter^*, PRNP*^+/Ter^, and *PRNP*^+/+^ goats were subjected to intracerebral inoculation with goat scrapie prions. Disease progression was studied by clinical and neurological examinations, as well as post-mortem histopathological, immunohistochemical, and gene expression analyses. Furthermore, a thorough longitudinal clinical evaluation provides information on the range of clinical signs in goat scrapie.

## Materials and methods

### Animals

Twelve female Norwegian dairy goats (4 *PRNP*^+/+^, 4 *PRNP*^+/Ter^, and 4 *PRNP*^Ter/Ter^) were recruited from a research herd at the Norwegian University of Life Sciences. The average kinship coefficient of included animals was 10.8% (Additional file 1). The mean age was 3.3 (SD = 0.5) months and the mean body weight was 18.4 (SD = 2.8) kg. The animals were kept under a 16 h light/8 h dark cycle and housed in three groups of 4 goats according to genotype. Hay and water were provided ad libitum, and they were provided with a commercial goat pellet concentrate twice a day. The goats were acclimatized in the new facilities for 14 days, and clinical examinations and hematology were performed to ensure healthy animals before inoculation.

### Inoculation procedure

The inoculum was derived from the brain of a 3.5-year-old goat with the wild-type *PRNP* genotype with natural clinical scrapie as described in [24]. The goats were inoculated intracerebrally with 400 µL of the goat scrapie brain inoculum (10% wt/vol). In brief, goats were anesthetized with Zoletil vet. (tiletamine and zolazepam, 5.5 mg/kg intramuscularly) and local anesthetics were administered subcutaneously. A midline incision was made in the skin at the junction of the parietal and frontal bones, and a 1 mm hole was drilled through the calvarium. The inoculum was injected into the midbrain via a 21G and 5 cm long needle while the needle was being withdrawn from the brain. The skin incision was closed with two sutures. The goats received a single dosage of flunixin meglumine (2.2 mg/kg) and antibiotics (procaine benzylpenicillin, 45 mg/kg) for 3 days.

### Sampling

Clinical and neurological examinations were performed approximately once a month from day 432 post-inoculation (dpi). The examination included assessment of behavior, cranial nerve function, proprioception, signs of pruritus (alopecia and scratch test), body condition score, and evaluation of locomotion in a corridor. The full neurological protocol was modified from [25] and can be found in Additional file 2. The groups of normal goats (*PRNP*^+/+^) and heterozygous goats (*PRNP*^+/Ter^) were euthanized when at least one animal in the group had advanced clinical signs of prion disease. The *PRNP*^Ter/Ter^ goats were euthanized approximately 1 year after the heterozygous group. Euthanasia was performed by an overdose of pentobarbital, and a full necropsy was performed. The left half of the brain, the whole spinal cord and cauda equina with corresponding dorsal root ganglia, and a range of peripheral organs were sampled. Tissue samples for histology and immunohistochemistry (IHC) were immersion-fixed in 4% formaldehyde for approximately 1 week, dehydrated in graded ethanol, and paraffin embedded. Tissues for enzyme immunoassay (EIA) and real-time quaking-induced conversion (RT-QuIC) were frozen at −70 °C until further processing. Samples for RNA extraction were collected from hippocampus within 20 min after euthanasia, and immediately immersed in RNA-later (Invitrogen, Oslo, Norway) and stored at −70 °C.

### Histopathology

The brain was sectioned at six levels: (1) the olfactory lobe; (2) section through the frontal cortex at the level of ansate sulcus; (3) transverse section through the piriform lobe and thalamus; (4) transverse section through the rostral aspect of the superior colliculus; (5) transverse section through the brainstem and cerebellum at the level of the caudal cerebellar peduncles; and (6) transverse section through the obex. In addition, one section of cervical, thoracic, and lumbar spinal cord and cauda equina with corresponding dorsal root ganglia were selected for analysis. Peripheral tissues included in this study were the trigeminal ganglion, parotideal-, medial retropharyngeal- and superficial cervical lymph nodes, spleen, adrenal gland, and mucosa-associated lymphoid tissue at the recto-anal border. From each of these tissues and CNS areas, 4 µm sections were stained with hematoxylin and eosin and evaluated. Serial sections for IHC were mounted on Superfrost^®^ Plus slides (Menzel-Gläser, Thermo Scientific, Oslo, Norway).

To generate a brain-lesion profile, vacuolation was graded from 0 to 5 as follows: 0, no vacuoles; 1, few vacuoles, unevenly distributed; 2, few vacuoles, evenly distributed; 3, moderate numbers of vacuoles, 10–20 per 40x-field; 4, many vacuoles, with tendency of coalescence; and 5, dense vacuolation with coalescence. The brain areas and scores were slightly modified from Fraser and Dickinson [26], since the original lesion profile is described in mice.

### Immunohistochemistry

Sections were dried overnight at 58 °C. All tissues listed above were PrP-immunolabelled with the monoclonal antibody F89 (Abcam, Cambridge, UK), dilution 1:2000. Glial fibrillary acidic protein (GFAP) labeling, dilution 1:1500 (Dako, Glostrup, Denmark, Z0334), was performed on sections including thalamus and hippocampus. The sections were deparaffinized in xylene and rehydrated through decreasing concentrations of graded ethanol. The protocol for PrP-immunolabeling included demasking steps in 98% formic acid for 5 min, followed by hydrated autoclaving in citrate buffer (pH 6.0) at 121 °C for 15 min. Endogenous peroxidase activity was blocked by incubation in 3% H_2_O_2_ in methanol for 20 min at room temperature. Sections were then blocked in normal goat serum (1:50) diluted in phosphate-buffered saline (PBS) for 20 min and incubated with the primary antibody for 1 h at room temperature. Further steps were performed with the EnVison+ System-HRP AEC (Dako, K4005, mouse or K4009, rabbit). Sections were counterstained in hematoxylin and mounted using Faramount medium (Agilent, Oslo, Norway). Washing between steps was performed with Tris-buffered saline (TBS). All runs included a negative control section where the primary antibody was replaced with 1% BSA, and a brain or lymph node section from a known scrapie-negative animal.

Sections were examined by light microscopy, and both PrP^Sc^ and GFAP signals were semi-quantitatively scored as follows: 0 = negative; 1 = sparse; 2 = moderate; 3 = marked, including half-step grading. Astrocyte GFAP evaluation also included scoring of the number and localization of cells, and the appearance of primary and secondary processes.

### IDEXX EIA test

Tissue homogenates (hippocampus, superficial cervical lymph node, and spleen) were prepared from 200 mg tissue and 1000 µL dH_2_O and analyzed with the HerdChek Scrapie/BSE Antigen EIA test (IDEXX, Hoofddorp, Netherlands) according to the manufacturer’s instructions. In brief, 120 µL brain homogenate (20% wt/vol) was mixed with 30 µL of the working plate diluent. For lymph node and spleen tissue, 100 µL homogenate (20% wt/vol) was mixed with 50 µL of the working plate diluent. One hundred microliter of the diluted sample was transferred onto the assay plate, incubated for 45 min with low shaking and washed six times. A conditioning buffer (100 µL) was added to each well and the plate was incubated for 10 min and then washed three times. The plate was incubated with a HRPO-conjugated anti-PrP antibody for 45 min, washed five times, before adding 100 µL tetramethylbenzidine substrate per well. After 15 min incubation, the reaction was stopped by adding 100 µL hydrochloric acid (1 M) and the absorbance was read at 450 nm and 620 nm. The cut-off value for a scrapie-positive result was calculated based on the following formula: the negative control mean (NCx̄) + 0.180.

### RT-QuIC analysis

Truncated Syrian hamster (90–231) recombinant PrP (SHrPrP) solution (purchased from Colorado State University, USA) was used as substrate for all samples. The substrate was thawed at room temperature and filtered through a 100 kDa Nanosep centrifugal device (Pall Corporation, New York, USA). The RT-QuIC reaction was performed by adding 2 µL of 10 ^−2^/10 ^−3^ diluted seeding samples (hippocampus, 20% wt/vol) to 98 µL reaction mix composed of 20 mM NaH_2_PO_4_ (pH 7.4), 320 mM NaCl, 1.0 mM EDTA, 10 µM Thioflavin T (ThT), 0.001% sodium dodecyl sulfate, 0.1% N_2_, and 0.04 mg/mL SHrPrP substrate in wells of a black 96-well plate with clear bottom. The plate was sealed with plate-sealer film (Nalgene Nunc International, Roskilde, Denmark) and incubated at 42 °C in a BMG FLUOstar Omega plate reader with cycles of 60 s shaking (700 rpm, double orbital) followed by 60 s of rest. In one sample with low prion levels (#536), longer shaking intervals (90 s shaking and 30 s rest) were used to promote faster RT-QuIC kinetics [27]. ThT measurements (450 ± 10 nm excitation and 480 ± 10 nm emission; bottom read; gain 1.700) in each well were read every 15 min, for a total of at least 60 h. Fluorescence values were plotted as the average of triplicate reactions versus incubation time (GraphPad software Inc, San Diego, USA). RT-QuIC reaction was classified as positive if the fluorescence of at least two out of three replicates exceeded a threshold determined to be 5 SD above the average baseline fluorescence.

### RNA extraction and cDNA synthesis

RNA was extracted from approximately 30 mg of tissue using the RNeasy Lipid Tissue Mini Kit (Qiagen, Hilden, Germany, 74804) according to the manufacturer’s instruction. The isolated RNA was quantified at optical density (OD)_260_ and purity was assessed by OD_260/280_ and OD_260/230_ absorbance readings with a DeNovix DS-11 spectrophotometer. RNA integrity (RIN) was assessed using the 2100 BioAnalyzer with RNA 6000 Nano kits (Agilent). Mean RIN value of included samples ± SD was 7.2 ± 0.30. Complementary DNA synthesis was performed using the QuantiTect Reverse Transcription Kit (Qiagen) according to the manufacturer’s instructions, with 600 ng RNA from each sample as templates. For each primer assay, a non-reverse transcriptase control was included to evaluate potential genomic DNA background signals, and a no template control was used to assess non-specific amplification or sample contamination.

### Quantitative PCR

The expression of seven target genes (*PRNP, GFAP, SAA3*, *CXCL10*, *CD14*, *S100A9*, and *IL1B*) was investigated by the LightCycler 480 quantitative PCR (qPCR) system. Primers were designed to span exon/exon boundaries and to cover known splice variants by the Primer3 software [28]. ACTB and SAA3 sequences were adapted from [29, 30]. Primer sequences are given in Additional file 3. Quantitative PCR reactions were performed using SYBR Green PCR Master Mix, including 10 µL cDNA (1:10) in each reaction and standard cycling conditions: initial denaturation for 5 min at 95 °C, followed by 42 amplification cycles (10 s at 95 °C, 10 s at 60 °C and 15 s at 72 °C) and final construction of melting curves. A standard curve was generated for each target gene to obtain primer amplification efficiencies, correlations, and dynamic range. Normalization was performed against the *ACTB* reference gene, and relative expression was calculated using the 2 ^−ΔΔCq^ method, as described in [31].

### Descriptive and statistical analysis

Data are presented as mean ± standard error of the mean (SEM). Graphical and statistical analyses were performed in GraphPad Prism 6 (GraphPad software Inc.). Associations between clinical signs and scrapie were analyzed by Barnard’s test for contingency tables. The Mann–Whitney *U* test was used to compare differences in ordinal data including the brain lesion profile and immunohistochemical scoring. Differences in gene expression were analyzed by one-way ANOVA with Tukey post hoc test for multiple comparisons. A *p* value < 0.05 was considered statistically significant.

## Results

### Clinical signs of prion disease are delayed in *PRNP*^+/Ter^ goats

**Figure 1:**
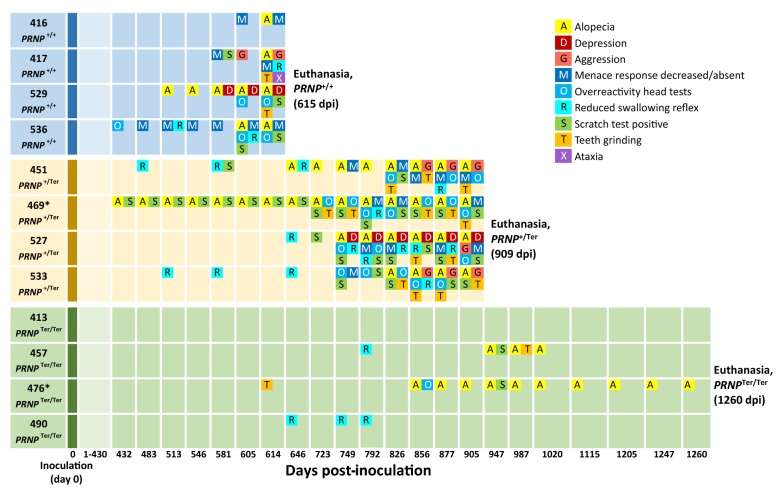
**Timeline of clinical and neurological signs.** Goats of three different genotypes (4 *PRNP*^+/+^, 4 *PRNP*^+/Ter^ and 4 *PRNP*^Ter/Ter^) were intracerebrally inoculated with goat scrapie prions at day 0. The figure illustrates the progression of nine selected clinical signs at the indicated days after inoculation (dpi). Neurological exams were performed in all animals at each time point until euthanasia. *Goat 469 had early signs of alopecia associated with an allergic dermatitis. Goat 476 had mild signs of alopecia, and generally a dull and flaking coat not related to scrapie.

Figure 1 summarizes the clinical progression of nine selected clinical signs in the 12 goats (4 *PRNP*^+/+^, 4 *PRNP*^+/Ter^ and 4 *PRNP*^Ter/Ter^) included in the study. The mean incubation period until clinical signs of prion disease was 601 dpi (SD = 14) in the *PRNP*^+/+^ goats. This group was euthanized 615 dpi, when goat #417 had developed advanced clinical signs dominated by aggressive behavior over a short period of time. The mean incubation period in the heterozygous group was 773 dpi (SD = 46) and the goats were euthanized 909 dpi. Goat #527 gradually developed depression, social withdrawal, alopecia, and pruritus before euthanasia. One heterozygous animal (#469) had early signs of alopecia from day 432 dpi and intermittent periods of pruritus, not associated with scrapie. Histopathological evaluation of skin biopsies indicated an allergic dermatitis, but the etiology was not identified. The progression of clinical signs was much slower in in *PRNP*^+/Ter^ goats than in *PRNP*^+/+^ goats (136 vs 14 days). The four *PRNP*^Ter/Ter^ goats remained healthy without any clinical signs of prion disease until they were euthanized 1260 dpi. However, one goat (#476) had mild signs of alopecia, and a dull and flaking coat throughout the last year. The complete open reading frame of *PRNP* was sequenced in all 12 goats to make sure that already-known polymorphisms associated with scrapie susceptibility would not bias the results. In addition to the stop-mutation in codon 32 of *PRNP*^Ter/Ter^ and *PRNP*^+/Ter^ goats, polymorphisms were observed at codon 220 and 240 (Additional file 4), but none of these have been reported to affect incubation times. Moreover, the high average kinship of the goats (Additional file 1) indicates that the *PRNP* genotype is the main factor causing the observed differences between the groups.

### Clinical signs are linked to scrapie

Table 1 shows the distribution of clinical signs in goats that were confirmed scrapie-positive (8 goats) or scrapie-negative (4 goats). At the time of euthanasia, all scrapie-positive goats displayed mild to moderate signs of alopecia. Various areas were affected, but most frequently were the poll (7 of 8), pelvic region (7 of 8), and neck (5 of 8). A positive scratch test, teeth grinding, and overreactivity of head tests were also significantly overrepresented in scrapie-positive goats. Aggressive behavior, such as biting towards the tail and perineal area and horning, was observed in 4 of 8 goats, whereas depression was observed in 2 of 8 goats.

**Table 1:** **Distribution of clinical signs observed within the last month before euthanasia**

Clinical sign	Scrapie-positive goats (*PRNP*^+/+^ and *PRNP*^+/Ter^)	Scrapie-negative goats (*PRNP*^Ter/Ter^)	Barnard’s test *p* value
Alopecia	8/8	1/4	0.0005
Scratch test, positive	7/8	0/4	0.006
Teeth grinding	6/8	0/4	0.019
Head tests, overreactivity	6/8	0/4	0.019
Menace response, decreased	5/8	0/4	NS (0.056)
Aggression	4/8	0/4	NS (0.100)
Swallowing reflex, reduced	4/8	0/4	NS (0.100)
Depression	2/8	0/4	NS (0.425)
Ataxia	1/8	0/4	NS (0.887)

NS: not significant.

### Histopathological changes

**Figure 2:**
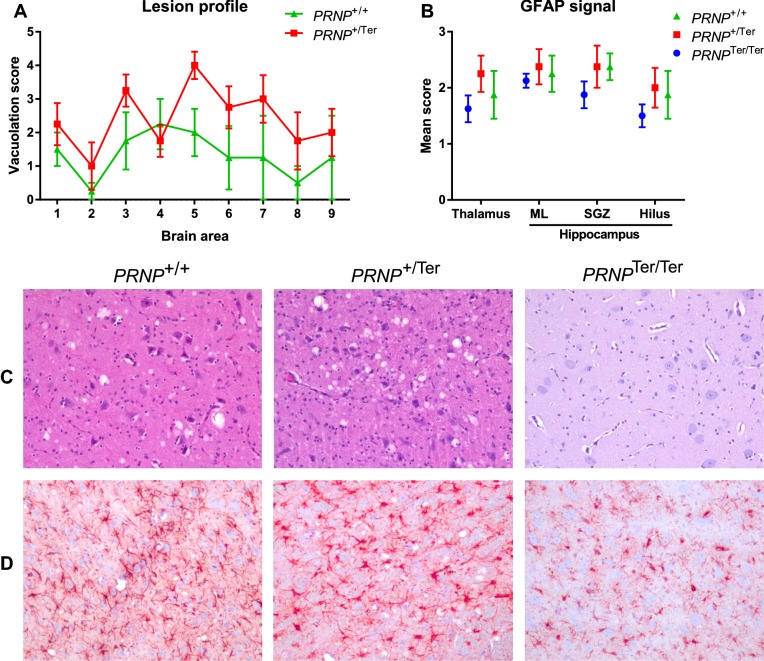
**Histopathological changes and GFAP immunohistochemistry.** **A** Vacuolation was graded 0–5 in nine defined brain areas (modified from Fraser and Dickinson 1968): 1, rostral part of medulla oblongata at the level of the obex; 2, transverse section of cerebellar cortex; 3, dorsal grey substance of the superior colliculus; 4, hypothalamus; 5, thalamus: massa intermedia and central nuclei; 6, hippocampus; 7, septal nuclei; 8, cerebral cortex dorsal to corpus callosum; and 9, cerebral cortex dorsal to septal nuclei. **B** GFAP signals were scored from 0 to 3 in thalamus and in three layers of the hippocampus (ML, molecular layer; SGZ, sub-granular zone; and hilus). **C** Vacuoles were primarily located in the neuropil of grey matter. Pictures show a representative vacuolation score 4 (*PRNP*^+/+^) and score 5 (*PRNP*^+/Ter^) in the thalamus. **D** Astrocyte GFAP labeling displayed increased length and thickening of primary and secondary processes in the thalamus of some scrapie-positive animals (goat #417 and #527). Values are mean ± SEM. *n* = 12 (4 *PRNP*^+/+^, 4 *PRNP*^+/Ter^, and 4 *PRNP*^Ter/Ter^). Magnification: **C**, **D** ×200.

Vacuolation was scored in nine defined brain regions to generate a lesion profile (Figure 2A). Although the mean vacuolation score was higher in *PRNP*^+/Ter^ goats than in *PRNP*^+/+^ goats in 8 of 9 brain regions, this difference was not statistically significant. As expected, the most severe changes were seen in the midbrain and thalamus (Figure 2C), near the inoculation site. The vacuoles were primarily associated with neuropil of grey matter and only a limited number of vacuoles were intraneuronal. Neuropil vacuolation was found throughout the spinal cord of both *PRNP*^+/Ter^ and *PRNP*^+/+^ goats, and the dorsal horn was more affected in both groups. Scar tissue associated with the inoculation, also macroscopically visible, was found in two *PRNP*^+/+^ animals and one *PRNP*^Ter/Ter^ animal.

No significant differences in the number of GFAP-positive astrocytes, or in the length and thickness of primary and secondary processes, were observed between genotypes (Figure 2B). Nevertheless, the two animals with the most severe clinical signs and vacuolation (#417 and #527) had longer and thicker astrocytic primary and secondary processes in the thalamus when compared with *PRNP*^Ter/Ter^ goats (Figure 2D and Additional file 5).

### Distribution of PrP^Sc^ in the CNS and peripheral tissues

**Figure 3:**
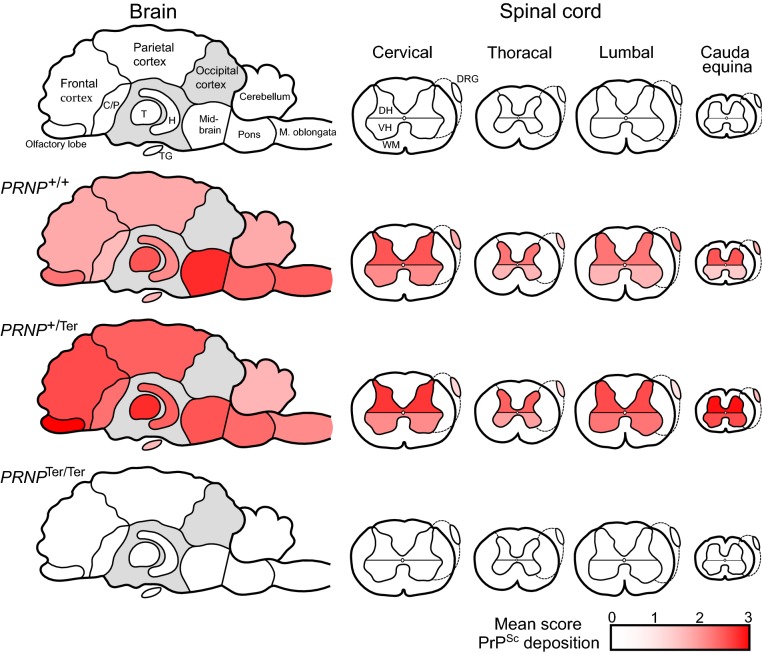
**Schematic overview of PrP**^**Sc**^ **deposition in the CNS and ganglia.** PrP^Sc^ was observed throughout the CNS and in dorsal root ganglia and trigeminal ganglia of scrapie-positive animals. Generally, the *PRNP*^+*/Ter*^ animals had slightly higher levels of PrP^Sc^ than *PRNP*^+/+^ animals in the cranial part of the brain and in the spinal cord. The *PRNP*^+/+^ goats had more PrP^Sc^ accumulation in the ganglia than the heterozygous goats. No PrP^Sc^ was observed in *PRNP*^Ter/Ter^ goats. Abbreviations: C/P, caudate nucleus and putamen; T, thalamus; H, hippocampus; TG, trigeminal ganglion; DH, dorsal horn; VH, ventral horn; WM, white matter; DRG, dorsal root ganglion. Graded red color indicates the magnitude of PrP^Sc^ accumulation, whereas grey color are brain regions that were not examined. This scheme summarizes the data described in Additional file 4.

**Figure 4:**
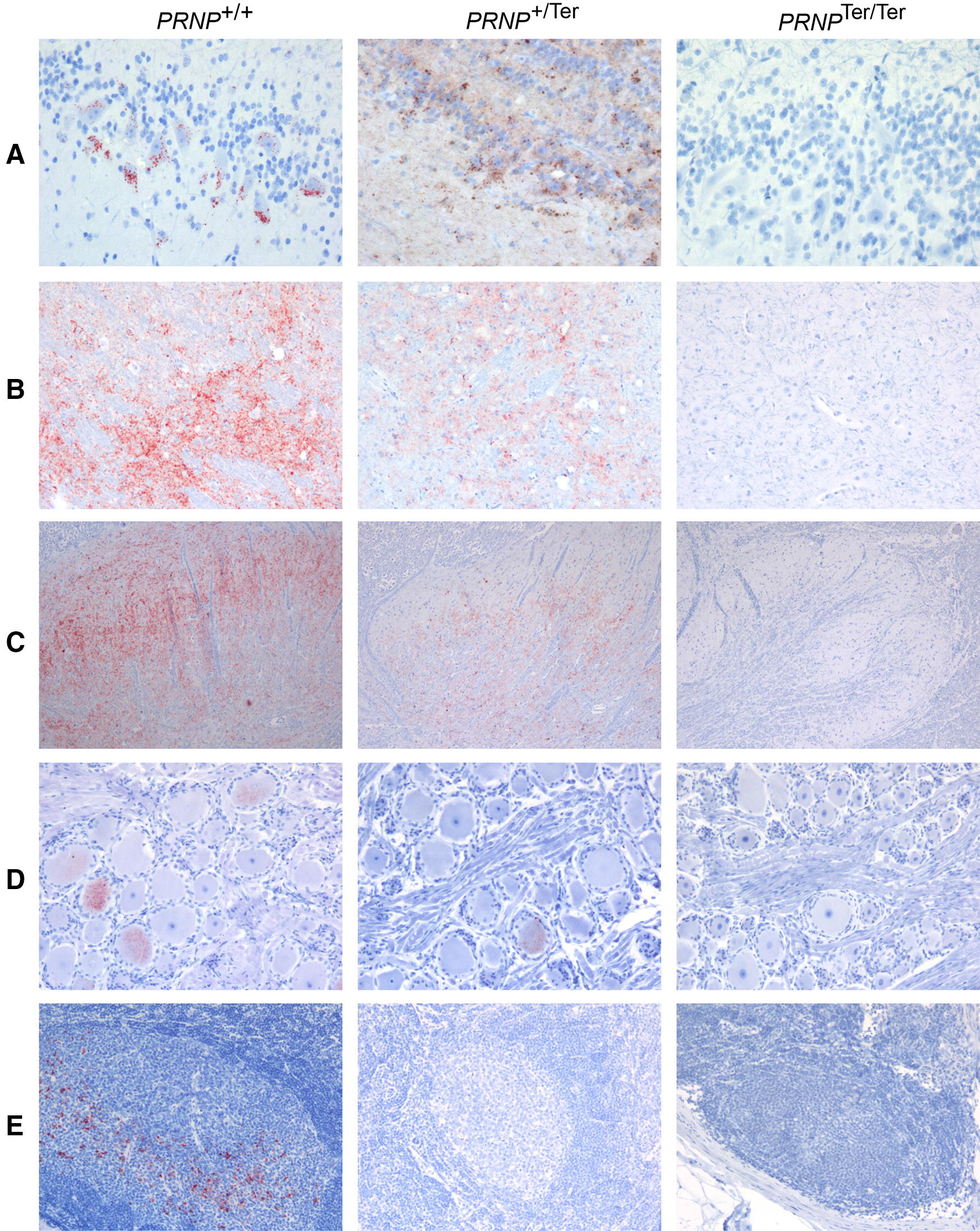
**PrP**^**Sc**^ **immunohistochemistry of the CNS and peripheral tissues.** **A** In the olfactory lobe, distinct granular PrP^Sc^ labeling of mitral cells was observed, but also neuropil and glial labeling involving the other layers was found. **B** In thalamus, there is moderate to marked PrP^Sc^ labeling that is mainly found in the neuropil. **C** There is marked PrP^Sc^ accumulation in the neuropil of the dorsal horn of both *PRNP*^+/+^ and *PRNP*^+/Ter^ goats. **D** There is sparse to moderate labelling of both neurons and surrounding satellite cells in the trigeminal ganglion. **E** A follicle in the retropharyngeal lymph node of a *PRNP*^+/+^ goat is PrP^Sc^ positive, while this lymph node was negative in the heterozygous group. **A**–**E** There was no accumulation of PrP^Sc^ in the CNS or peripheral tissues in any of the *PRNP*^Ter/Ter^ goats. Magnification: **A** ×400; **B**, **D**, **E** ×200; **C** ×100.

The distribution of PrP^Sc^ in the CNS (Figure 3) correlated with vacuolation and was similar in the *PRNP*^+/+^ and *PRNP*^+/Ter^ goat groups, reaching the olfactory lobe (Figure 4A) cranially and cauda equina caudally. The animals with the mildest clinical signs, such as goat #416, also had less severe pathology and accumulation of PrP^Sc^ (Additional file 6).

Generally, neuropil deposits of PrP^Sc^ dominated, with less intraneuronal and intraglial labeling. The highest levels of PrP^Sc^ deposition were observed in brain regions near the area of inoculation (Figure 4B). In three of the heterozygous animals, fibrillar PrP^Sc^-positive plaques were observed in several brain areas. PrP^Sc^ accumulation was observed throughout the spinal cord, more pronounced in the dorsal than in the ventral horns (Figure 4C). No PrP^Sc^ labeling of white matter occurred in the spinal cord. In the dorsal root ganglia and trigeminal ganglia there was sparse to moderate labeling of both neurons and satellite cells (Figure 4D). Nerve fibers in these ganglia were not labeled.

All four *PRNP*^+/+^ goats were positive in the retropharyngeal lymph node, but only in a few or moderate number of follicles (Figure 4E). There were also a few positive cells in sinuses associated with the positive follicles. In contrast, none of the *PRNP*^+/Ter^ animals were positive in the retropharyngeal lymph node, but one heterozygous goat was positive in the parotideal lymph node (Additional file 6). In this animal, positive cells, most likely macrophages, were found in the capsular sinus, and associated with positive follicles. Unfortunately, this lymph node was not sampled in the *PRNP*^+/+^ goats. All animals were negative in the superficial cervical lymph nodes, rectoanal mucosa-associated lymphoid tissue, spleen, and adrenal glands.

There was no accumulation of PrP^Sc^ in the CNS or peripheral tissues in any of the *PRNP*^Ter/Ter^ goats (Figures 3 and 4A–E). Individual scoring-data of PrP^Sc^ in the CNS and peripheral tissues can be found in Additional file 6.

### Validation of PrP^Sc^-immunohistochemistry by EIA and RT-QuIC

**Figure 5:**
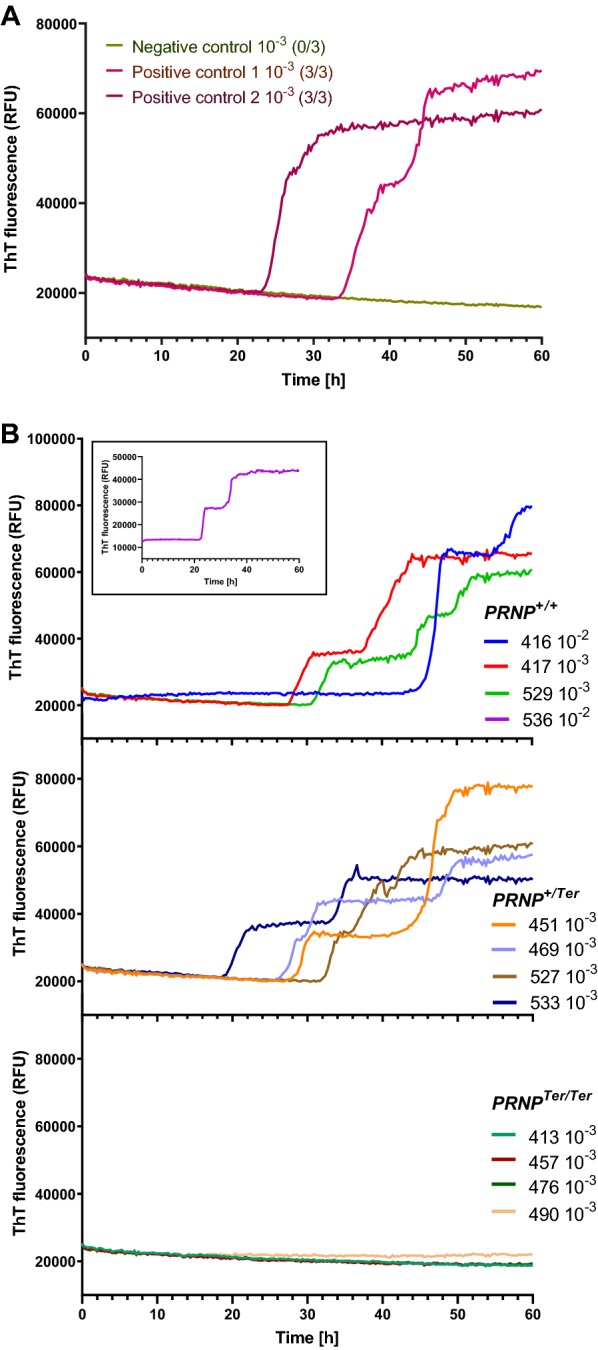
**Detection of prion seeding activity by RT-QuIC.** RT-QuIC was performed using SHrPrP (90–231) as substrate. **A** Brain homogenate from two sheep inoculated with classical scrapie (positive control 1 and 2) and homogenate from a known scrapie-negative sheep (negative control) were used to assess the overall performance of the RT-QuIC. **B** Hippocampal homogenates diluted at 10 ^−2^ or 10 ^−3^ were analyzed as described in materials and methods. Inserted graph shows amplification of sample #536 at a slightly modified experimental protocol. Samples were analyzed in triplicates and the curves show the average fluorescence intensity at each time point.

There was a high similarity between the results of the three methods used for detection of prions: IHC, EIA and RT-QuIC (Table 2). Sections from hippocampus (#416 and #536) with a low IHC score (0.5), also had lower EIA absorbance values than sections with a higher IHC score (> 1.5). Peripheral tissues, including the spleen and superficial cervical lymph node, were confirmed negative by EIA in all animals. The RT-QuIC method was optimized by running samples (brain and lymphatic tissues) from sheep inoculated with classical scrapie. Homogenate from a known scrapie-negative sheep was included as negative control (Figure 5A). Different dilutions (10 ^−2^ to 10 ^−5^) of brain homogenate were analyzed, of which the 10 ^−2^/10 ^−3^ dilutions were considered optimal for detecting an increase in ThT fluorescence. Hippocampal prion seeding activity was detected in at least 2 of 3 replicates from all scrapie-positive goats (Figure 5B). All samples from *PRNP*^Ter/Ter^ goats were confirmed scrapie-negative by EIA and RT-QuIC.

**Table 2:** **Comparison of the diagnostics tests IHC, EIA and RT-QuIC on selected tissues**

Genotype	Goat#	Hippocampus			SCLN		Spleen	
		IHC^a^	EIA^b^	RT-QuIC^c^	IHC^a^	EIA^b^	IHC^a^	EIA^b^
*PRNP*^+/+^	416	0.5	2.286	3/3	0.0	–	0.0	–
*PRNP*^+/+^	417	3.0	> 3.50	3/3	0.0	–	0.0	–
*PRNP*^+/+^	529	1.5	> 3.50	3/3	0.0	–	0.0	–
*PRNP*^+/+^	536	0.5	0.594	2/3	0.0	–	0.0	–
*PRNP*^+/Ter^	451	2.5	> 3.50	3/3	0.0	–	0.0	–
*PRNP*^+/Ter^	469	1.5	> 3.50	3/3	0.0	–	0.0	–
*PRNP*^+/Ter^	527	2.0	> 3.50	3/3	0.0	–	0.0	–
*PRNP*^+/Ter^	533	1.5	> 3.50	2/3	0.0	–	0.0	–
*PRNP*^Ter/Ter^	413	0.0	–	0/3	0.0	–	0.0	–
*PRNP*^Ter/Ter^	457	0.0	–	0/3	0.0	–	0.0	–
*PRNP*^Ter/Ter^	476	0.0	–	0/3	0.0	–	0.0	–
*PRNP*^Ter/Ter^	490	0.0	–	0/3	0.0	–	0.0	–

SCLN, superficial cervical lymph node; IHC, immunohistochemistry; EIA, enzyme immunoassay; RT-QuIC, real-time quaking-induced conversion.

^a^Immunohistochemical score of PrP^Sc^ from 0 to 3.

^b^IDEXX enzyme immunoassay absorbance values (A_450_). Negative results (−) are below cut off: 0.198. All negative samples were below 0.033.

^c^RT-QuIC results show positive replicates/total number of replicates.

### Gene expression analysis

**Figure 6:**
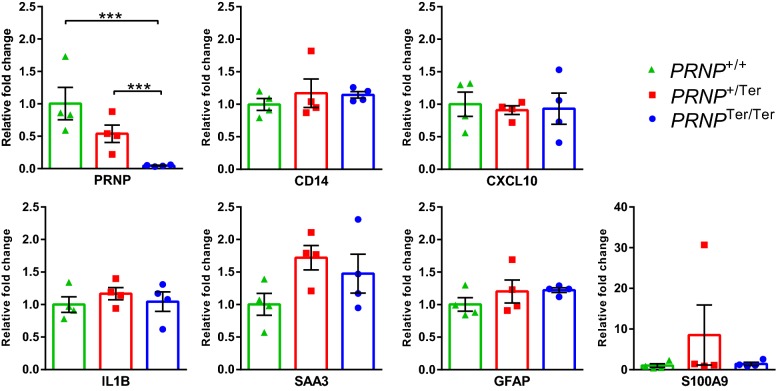
**Relative gene expression in hippocampus at the time of euthanasia.** Gene expression of seven target genes was measured by qPCR. For each gene, the expression levels are normalized relative to the *PRNP*^+/+^ group (= 1). Bars display mean expression (± SEM) with asterisks representing significant differences (*p* < 0.001) assessed by one-way ANOVA with Tukey post hoc test. *n* = 12 (4 *PRNP*^+/+^, 4 *PRNP*^+/Ter^, and 4 *PRNP*^Ter/Ter^).

The expression of *PRNP* and six selected genes (*GFAP*, *SAA3*, *CXCL10*, *CD14*, *S100A9*, and *IL1B*) associated with inflammation and stress response was investigated in hippocampal tissue (Figure 6). The genes were selected based on differential expression in different scrapie models [32–35]. The levels of *PRNP* correlated with genotype and was 54% in *PRNP*^+/Ter^ goats and about 5% in *PRNP*^Ter/Ter^ goats compared with *PRNP*^+/+^ goats. None of the other six target genes were differentially expressed between genotypes. However, goat #527, which was most clinically affected, had increased expression levels of GFAP and S100A9.

## Discussion

In this study we show that goats naturally devoid of PrP^C^ do not replicate prions and therefore are resistant to prion diseases. The *PRNP*^+/+^ goats were euthanized at 615 dpi, when clinical signs of scrapie were observed in all four animals in the group. Histopathological examination showed vacuolation and accumulation of PrP^Sc^ in the CNS of all four goats, which confirms that the goat scrapie inoculum was consistently infectious. In contrast to the *PRNP*^+/+^ goats, none of the *PRNP*^Ter/Ter^ goats had clinical signs of scrapie or evidence of PrP^Sc^ accumulation at 1260 dpi, which confirms that the *PRNP*^Ter^-mutation confer resistance against scrapie in animals without PrP^C^. These results are in accordance with PrP^Sc^ inoculation of genetically modified *Prnp*-knock out animals, which are unable to replicate prions and do not develop prion disease [3, 4].

Heterozygous goats had, on average, 29% longer incubation time (773 versus 601 days) and a much slower progression in the development of clinical signs than *PRNP*^+/+^ goats. Interestingly, inoculation of heterozygous (*Prnp*^0/+^) mice with RML mouse-adapted scrapie resulted in a 70 to 120% increase in incubation time when compared with wild type mice [36–38]. This suggests that the protective effect of *PRNP*^Ter^ heterozygosity in goats, which is a natural host for scrapie, seems lower than expected based on the results from laboratory rodents. Studies of transgenic mice carrying different copy numbers of *Prnp* have shown that time to onset of clinical disease is inversely correlated with PrP^C^ expression [38, 39]. This is probably because neurotoxic PrP-isoforms accumulate in a process that is rate limited by, and directly proportional to, PrP^C^ concentrations [39, 40]. In addition, it has been demonstrated that neuronal expression of PrP^C^ is essential to mediate neurotoxic effects of PrP^Sc^ oligomers [5–7]. In the present study, hippocampal *PRNP* levels in heterozygous goats were 54% of those of *PRNP*^+/+^ goats, which is in line with studies of PrP^C^ levels in heterozygous animals [23, 39]. Thus, the delayed onset and slower progression of clinical disease in these animals probably reflect a combination of reduced availability of PrP^C^ as a substrate for prion replication, in addition to less PrP^C^ at the cell surface to mediate the toxic effects.

Although intracerebral inoculation does not mimic the natural oral route of prion infection, the current study provides insights into the spread of prions within the CNS and the centrifugal spread to peripheral tissues. Not surprisingly, the severity of histopathological changes and PrP^Sc^ accumulation was centered on the site of inoculation and spread cranially and caudally throughout the CNS. The distribution in the brain was similar to previous descriptions in goats and sheep upon intracerebral inoculation with scrapie [13, 41]. Interestingly, the severity of histopathological changes and PrP^Sc^ accumulation did not differ between the cervical region of the spinal cord and caudal segments, including cauda equina. As the inoculation site was near the ventricular system, one possible explanation could be that infectious prions were spread in the cerebrospinal fluid (CSF) during and after inoculation. Notably, high levels of prions have been observed in the CSF already at day 1 after intracerebral inoculation of hamsters [42]. In addition, neuronal spread likely contributed to the distribution of PrP^Sc^ within the CNS. We found only a limited centrifugal spread of prions to peripheral tissues, and positive tissues were either closely associated with the CNS, such as the dorsal root and trigeminal ganglia, or were head lymph nodes. If prions enter the CSF, it is likely that the meningeal lymphatic system [43, 44] could participate in transmission of PrP^Sc^ to head lymph nodes. The limited spread of PrP^Sc^ to peripheral organs, contrasts to some extent similar studies [13, 24]. The discrepancies could be due to differences in prion strains used for inoculation or associated with host factors, such as goat breed and genetic background. Whereas accumulation of PrP^Sc^ in the CNS did not differ between the genotypes, PrP^Sc^ was detected in head lymph nodes in all *PRNP*^+/+^ goats but only in 1 of 4 *PRNP*^+/Ter^ animals. Since levels of PrP^C^ is much higher in the CNS than in the periphery [45], it is possible that the amount of PrP^C^ is a more critical factor for prion replication in peripheral tissues than in the CNS. Thus, it would be of great interest to investigate scrapie-resistance in *PRNP*^+/Ter^ animals upon oral inoculation, since *PRNP*-expression in gut-associated lymphatic tissue is obligate for transmission of scrapie under natural conditions [46].

Goats without PrP^C^ could be useful as breeding goats to avoid scrapie in endemic regions and might also be valuable for production of “prion-free” bio-products, such as vaccines and antibodies. Such use would require animals with good general health and production parameters, and adverse effects due to lack of PrP^C^ should be ensured. During the last decades, substantial work has been directed towards understanding the normal function of PrP^C^. For instance, PrP^C^ is abundantly expressed in the male reproductive system [47, 48], and loss of PrP^C^ resulted in murine spermatozoa being more susceptible to Cu^2+^-induced stress [49]. To date, *PRNP*^Ter/Ter^ goats appear healthy under normal herd conditions [21] and detailed analyses of spermatozoa, both at rest and under acute stress, have not detected any abnormalities [50]. This suggests that *PRNP*^Ter/Ter^ goat bucks have normal sperm quality and fertility. In a study of acute systemic inflammation, however, goats without PrP^C^ displayed a prolonged sickness behavior [51] and increased activation of genes encoding for pro-inflammatory cytokines in the lungs [52]. This indicates that PrP^C^ could have a protective role against inflammatory stress [Reviewed in 22], and that *PRNP*^Ter/Ter^ goats might be more susceptible to infections. Larger epidemiological studies would be necessary to investigate the potential loss-of function phenotypes in *PRNP*^Ter/Ter^ goats in conjunction with important production-related diseases, such as mastitis and pneumonia. A possible drawback with breeding for the *PRNP*^Ter^-mutation is the modest protection against scrapie observed in heterozygous animals, at least upon intracerebral inoculation. In addition, the mutation has only been observed in Norwegian dairy goats, which limits the availability of breeding animals. Nevertheless, two other *PRNP* polymorphisms, S146 and K222, have been identified to confer strong resistance towards scrapie in goats, even in heterozygous animals [13–15, 53–55]. Although the allelic variants S146 and K222 are commonly found in many goat breeds, there is a wide variation in allele frequency across countries and regions [16–18]. Recently, a breeding model for scrapie resistance was carried out in two European dairy breeds reported to have low K222 allele frequencies [56]. The authors concluded that breeding for scrapie resistance can be implemented in goats, even though the rate at which resistant animals increased was slow.

Although prion infection does not induce a proper immune response, cells of the innate immune system have proven to be critical players in the initial pathogenesis of prion disease [57]. Moreover, activated glial cells release pro-inflammatory cytokines and chemokines that probably contribute to the disease development [58]. In the current study, none of the target genes (*GFAP*, *SAA3*, *CXCL10*, *CD14*, *S100A9*, and *IL1B*) associated with the acute phase response and inflammation were differentially expressed in the hippocampus of animals with and without scrapie. In contrast, previous inoculation studies in sheep and rodents have reported several differentially expressed genes in the brain [32–35]. The discrepancies could reflect differences in timing of euthanasia and thus the magnitude of neuropathology and inflammation in the brain. For example, gliosis and increased GFAP were observed in the terminal stage of a mouse scrapie model, but not in the earlier stages [33]. In the present study, increased *GFAP* expression was only observed in goat #527, which also exhibited profound clinical signs. Taken together, these findings underline the difficulty of identifying expression-based biomarkers of early prion disease in small ruminants.

In conclusion, our findings confirm that goats naturally devoid of PrP^C^ do not replicate prions and are therefore resistant to prion disease. The onset of disease and the progression of clinical signs is delayed in heterozygous animals. Pathological changes and the distribution of PrP^Sc^ in the CNS were similar in *PRNP*^+/+^ and *PRNP*^+/Ter^ goats, and there was only a limited peripheral spread of PrP^Sc^ to head lymph nodes and nervous tissue in close proximity to the CNS.

## Supplementary information

**Additional file 1. Kinship and degree of inbreeding.**

**Additional file 2. Neurological examination of goats.**

**Additional file 3. Primer sequences used for qPCR analysis.**

**Additional file 4. Sequence alignment of** ***PRNP*** **in 12 goats.**

**Additional file 5. Semi-quantitative scoring of GFAP.**

**Additional file 6. Distribution of PrP** ^**Sc**^ **in the CNS and peripheral tissues.**
