# Supplementary material for: Goats naturally devoid of PrPC are resistant to scrapie
Source: Vet Res. 2020 Jan 10;51:1. doi: 10.1186/s13567-019-0731-2 (PMC6954626; doi:10.1186/s13567-019-0731-2)
Supplement: Supplementary file 1 — Additional file 1. Kinship and degree of inbreeding. [file 13567_2019_731_MOESM1_ESM.pdf]

Kinship matrix (coefficients of relatedness)

Goat	417	529	536	451	469	527	533	413	457	476	490
**416**	0,3036	0,1269	0,1143	0,0361	0,0286	0,0888	0,0242	0,1476	0,0235	0,0163	0,0239
**417**		0,1028	0,1152	0,0381	0,0341	0,0944	0,0303	0,0910	0,0258	0,0218	0,0314
**529**			0,0858	0,1124	0,1236	0,0836	0,0930	0,2148	0,1006	0,0243	0,0553
**536**				0,0607	0,0718	0,2817	0,1269	0,0758	0,0448	0,0906	0,1032
**451**					0,3512	0,0977	0,3019	0,0374	0,2980	0,1451	0,0239
**469**						0,1231	0,3053	0,0367	0,3145	0,1539	0,0348
**527**							0,1004	0,0557	0,0819	0,0904	0,0351
**533**								0,0313	0,2924	0,2166	0,0987
**413**									0,0336	0,0258	0,0494
**457**										0,1795	0,0690
**476**											0,2682

Goat	Inbreeding coefficient
416	0.03322
417	0.00388
529	0.07347
536	0.01730
451	0.05512
469	0.06789
527	0.02739
533	0.01435
413	0.00701
457	0.01873
476	0.00198
490	0.02062

**Average coefficients of relatedness:**		%
All	0,108	**10,8**
Normal +/+	0,141	14,1
Heterozygotic +/Ter	0,213	21,3
Homozygotic Ter/Ter	0,104	10,4
Normal - heterozygotic	0,083	8,3
Normal - homozygotic	0,068	6,8
Heterozygotic - Homozygotic	0,122	12,2

Range: 0,016 - 0,35 (1,6 - 35 %)
