# Supplementary material for: Goats naturally devoid of PrPC are resistant to scrapie
Source: Vet Res. 2020 Jan 10;51:1. doi: 10.1186/s13567-019-0731-2 (PMC6954626; doi:10.1186/s13567-019-0731-2)
Supplement: Supplementary file 2 — Additional file 2. Neurological examination of goats. [file 13567_2019_731_MOESM2_ESM.pdf]

# NEUROLOGICAL EXAMINATION OF GOATS

**DATE:**

**ANIMAL NO:**

(✓ if normal , × if not able to observe, describe in detail if abnormal)

| | | | |
|---|---|---|---|
| 1. **Posture / Head Carriage** | | | |
| 2. **Abnormal Movements / Tremors / Fasciculations** | **None** ☐ / **Yes** ☐ (describe): | | |
| 3. **Mental Status** | | | |
| 4. **Behaviour** | **Resting (left alone):** | **Approached:** | **Handled:** |
| **FACE / NECK** | **Symmetry** (tick if all normal, or describe) | **Left** | **Right** |
| 5. **Olfaction (CN I)** | | | |
| 6. **Vision (CN II)** | | | |
| 7. **Eye position (CN III, IV, VI)** | | | |
| 8. **Menace response (CN II ++)** | | | |
| 9. **Palpebra reflex (CN V, CN VII)** | | | |
| 10. **Ears** (position/reaction to touch) | | | |
| 11. **Lips (CN VII)** (reaction to touch) | | | |
| 12. **Nose (CN VII) (**reaction to touch) | | | |
| 13. **Hearing (CN VIII)** | | | |
| 14. **Swallow (CN IX, CN X)** (on palpation) | | | |
| 15. **Jaw position, tongue tone (CN XII)** | | | |
| 16. **Withdrawal reflex** | | | |
| 17. **Salivation, dribbling** | **None** ☐ / **Yes** ☐ (describe): | | |
| 18. **Scratch/Nibble Test - Lumbar** | **Negative** ☐ / **Inconclusive** ☐ / **Inconsistent** ☐ / **Positive** ☐ | | |
| 19. **Body condition** | | **Body condition score:** | |

20. **Skin lesions: No** ☐ / **Yes** ☐ **-** list areas and types*/ draw below*

21. **Wool - hair loss: No** ☐ / **Yes** ☐ **-** list areas and types*/ draw below*

22. **Other Fleece change(s): No** ☐ / **Yes** ☐ **-** list areas and types:
*also draw below* (soiling/ changed fleece colour/ quality)

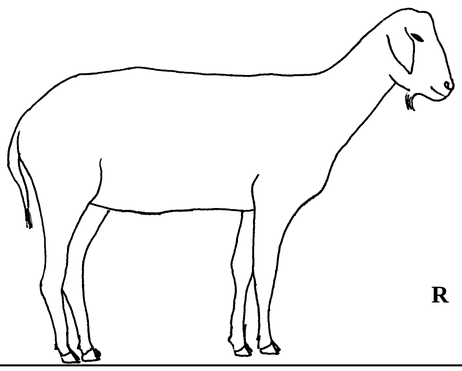

**R**

**L**

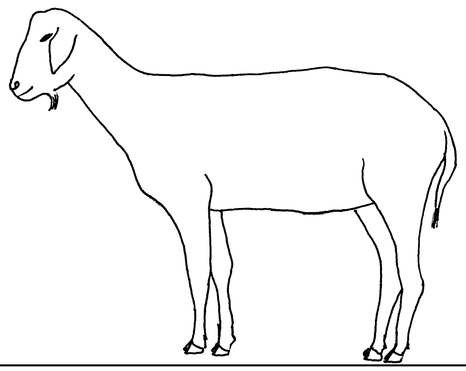

**GENERAL EXAMINATION**

23. **Temperature:** 24. **Heart rate:**

**GAIT**

| | |
|---|---|
| 25. **Walking** (amount/willingness) | |
| 26. **Running** (amount/ willingness) | |
| 27. **Slipping** | **No** ☐ / **Yes** ☐ (# of times, describe): |
| 28. **Falling** | **No** ☐ / **Yes** ☐ (# of times, describe): |
| 29. **Collides into walls** | **No** ☐ / **Yes** ☐ (# of times, describe): |

| | |
|---|---|
| *OVERALL GAIT*: | |
| 30. **Stiff / lame** | **No** ☐ / **Yes** ☐ – describe: |
| 31. **Neurological** | **No** ☐ / **May be** ☐ / **Yes** ☐ – describe: |
| 32. **Other on gait** | **No** ☐ / **Yes** ☐ – describe: |

33. **STATUS WITH REGARDS TO TSE & OTHER DISEASES:**

- no evidence of TSE ☐ Normal Animal ☐ / Other abnormality (ies) ☐
- suspect TSE ☐ → severity of case: early ☐ - established ☐ - advanced ☐
  → list suggestive signs (by their # if many):
- inconclusive with regards to TSE ☐ , detail:

**HISTORY:**

| **ACTION** | **DATE COMPLETED** | **RESULT** |
|---|---|---|
| Still photo: **No** ☐ / **Yes** ☐ (describe): | | |
| Video: **No** ☐ / **Yes** ☐ (describe): | | |
| Skin scraping: **No** ☐ / **Yes** ☐ (describe): | | |
| Other (describe): ______________________________ | | |

**Other Comments:**
