# Supplementary material for: Goats naturally devoid of PrPC are resistant to scrapie
Source: Vet Res. 2020 Jan 10;51:1. doi: 10.1186/s13567-019-0731-2 (PMC6954626; doi:10.1186/s13567-019-0731-2)
Supplement: Supplementary file 3 — Additional file 3. Primer sequences used for qPCR analysis. [file 13567_2019_731_MOESM3_ESM.pdf]

Quantitative PCR primer sequences

Gene ID	Symbol	Gene name	Primer sequences
102179831	ACTB	Actin beta	F: 5’TGCCCTGAGGCTCTCTTCCA<br>R: 5’TGCGGATGTCGACGTCACA
102169975	PRNP	Prion protein	F: 5’GTGGCTACATGCTGGGAAGT<br>R: 5’AGCCTGGGATTCTCTCTGGT
100860873	CXCL10	C-X-C motif chemokine ligand 10	F: 5’ACGCTGTACCTGCATCGAG<br>R: 5’GCAGGATTGACTTGCAGGA
102168428	SAA3	Serum amyloid A3	F: 5’CTGGGCTGCTAAAGTGATCAGTAAC<br>R: 5’CCCTTGAGCAGAGGGTCTGTGATT
102182273	S100A9	S100 calcium-binding protein A9	F: GAGATCATGGAGGACCTGGA<br>R: GGCCACCAGCATAATGAACT
102177056	CD14	Cluster of differentiation 14	F: CCACCCTCAGTCTCCGTAAC<br>R: GTGTGCTTGGGCAATGTTC
100860816	IL1B	Interleukin 1 beta	F: GACAACAAGATTCCTGTGGCC<br>R: TCTACTTCCTCCAGATGAAGTGT
102190069	GFAP	Glial fibrillary acidic protein	F: CAGATCCGAGGGGGCAAAAG<br>R: GGGGCTCCATCTACAACCTG
