# Supplementary material for: Goats naturally devoid of PrPC are resistant to scrapie
Source: Vet Res. 2020 Jan 10;51:1. doi: 10.1186/s13567-019-0731-2 (PMC6954626; doi:10.1186/s13567-019-0731-2)
Supplement: Supplementary file 4 — Additional file 4. Sequence alignment of PRNP in 12 goats. [file 13567_2019_731_MOESM4_ESM.pdf]

Sequence alignment of PrP^C in 12 goats. The 4 *PRNP*^Ter/Ter goats are homozygous for the Stop-mutation in codon 32, whereas the 4 *PRNP*^+/Ter goats are heterozygous (G/Ter). Some animals had polymorphisms in codon 220 and codon 240 (see table). *Capra hircus* prion protein (gi328682847) are shown as reference sequence.

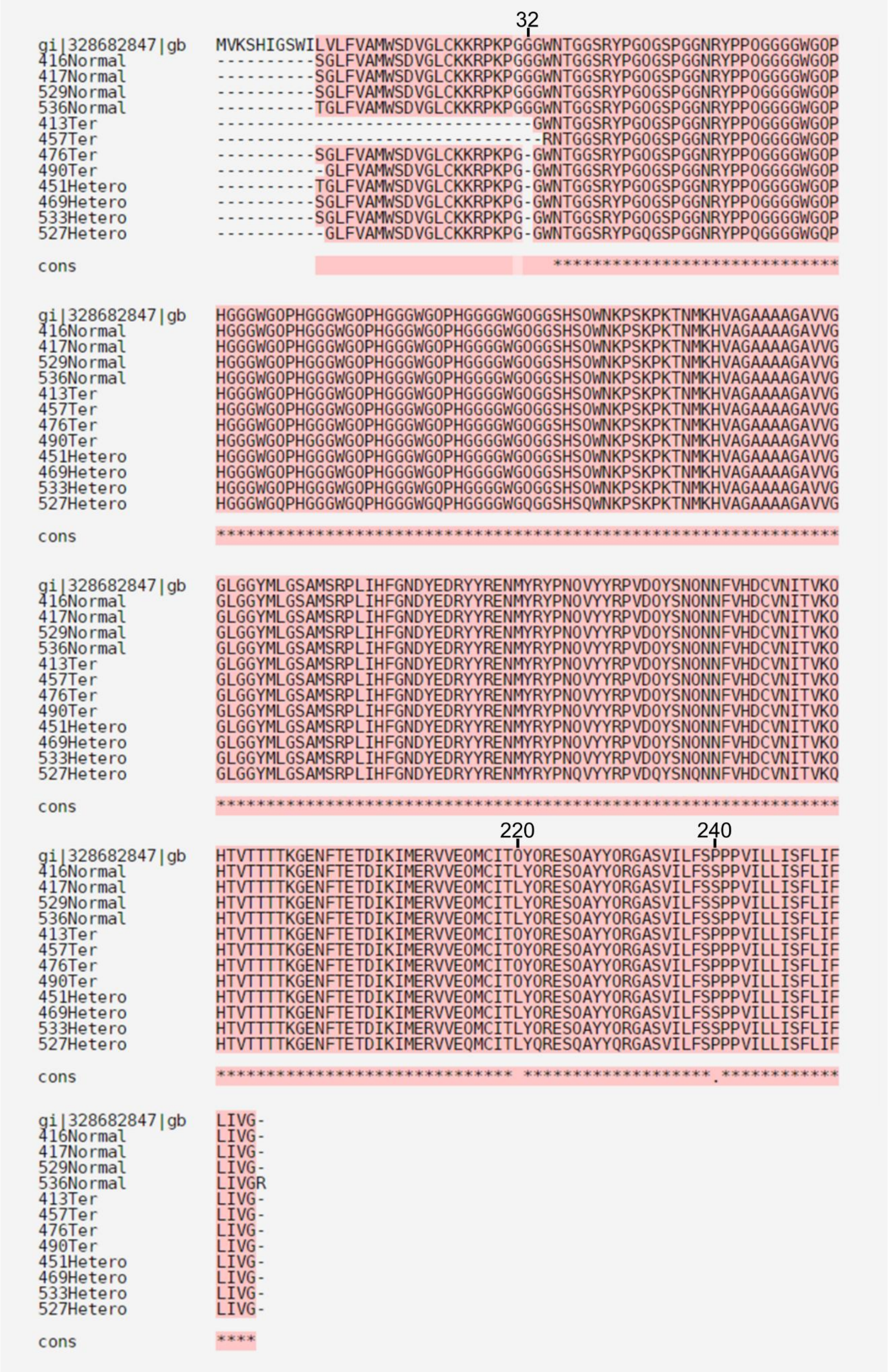

Overview of *PRNP* polymorphisms in 12 goats.

Goat#	32	220	240
416	G/G	L/L	P/P
417	G/G	L/L	P/P
529	G/G	L/L	P/P
536	G/G	L/L	P/P
413	Ter/Ter	Q/Q	P/P
457	Ter/Ter	Q/Q	P/P
476	Ter/Ter	Q/Q	P/P
490	Ter/Ter	Q/Q	P/P
451	G/Ter	Q/L	P/P
469	G/Ter	Q/L	P/P
533	G/Ter	Q/L	S/S
527	G/Ter	Q/L	P/S
