# Supplementary material for: Goats naturally devoid of PrPC are resistant to scrapie
Source: Vet Res. 2020 Jan 10;51:1. doi: 10.1186/s13567-019-0731-2 (PMC6954626; doi:10.1186/s13567-019-0731-2)
Supplement: Supplementary file 5 — Additional file 5. Semi-quantitative scoring of GFAP. [file 13567_2019_731_MOESM5_ESM.pdf]

Semi-quantitative scoring of GFAP-positive astrocytes in thalamus and hippocampus

					Hippocampus								
		Thalamus			Molecular layer			Sub-granular zone			Hilus		
**Genotype**	**Goat#**	S	1°	2°	S	1°	2°	S	1°	2°	S	1°	2°
PRNP$^{+/+}$	416	1.0	2.0	1.5	2.0	1.5	1.0	2.0	2.0	1.0	1.0	1.5	1.0
PRNP$^{+/+}$	417	3.0	3.0	2.0	2.5	2.5	2.0	2.5	2.5	1.5	2.0	2.5	2.0
PRNP$^{+/+}$	529	2.0	2.0	1.5	3.0	3.0	2.5	3.0	3.0	3.0	3.0	3.0	3.0
PRNP$^{+/+}$	536	1.5	1.5	1.0	1.5	1.5	1.0	2.0	2.0	1.5	1.5	2.0	2.0
		1.9	2.1	1.5	2.3	2.1	1.6	2.4	2.4	1.8	1.9	2.3	2.0
PRNP$^{+/Ter}$	451	2.5	3.0	2.0	2.5	1.5	1.5	2.0	2.0	1.5	2.0	3.0	2.0
PRNP$^{+/Ter}$	469	1.5	2.0	1.5	2.5	2.5	2.0	3.0	2.5	2.5	2.5	2.5	2.5
PRNP$^{+/Ter}$	527	3.0	3.0	2.0	3.0	2.5	2.5	3.0	2.5	2.5	2.5	2.0	2.0
PRNP$^{+/Ter}$	533	2.0	2.0	2.0	1.5	2.0	2.0	1.5	1.5	1.5	1.0	0.5	0.5
		2.3	2.5	1.9	2.4	2.1	2.0	2.4	2.1	2.0	2.0	2.0	1.8
PRNP$^{Ter/Ter}$	413	1.5	1.5	1.0	2.0	2.5	2.0	2.5	2.5	2.0	2.0	2.5	2.0
PRNP$^{Ter/Ter}$	457	2.0	1.5	1.0	2.0	1.5	1.0	1.5	2.0	1.0	1.5	1.0	1.5
PRNP$^{Ter/Ter}$	476	1.0	1.0	1.0	2.5	2.0	2.0	1.5	1.5	1.0	1.0	1.5	1.0
PRNP$^{Ter/Ter}$	490	2.0	3.0	2.5	2.0	1.5	1.5	2.0	2.0	2.0	1.5	1.5	1.5
		1.6	1.8	1.4	2.1	1.9	1.6	1.9	2.0	1.5	1.5	1.6	1.5

Abbreviations: S, mean GFAP signal; 1°, primary processes length and thickness; 2°, secondary processes length and thickness
