# Supplementary material for: Goats naturally devoid of PrPC are resistant to scrapie
Source: Vet Res. 2020 Jan 10;51:1. doi: 10.1186/s13567-019-0731-2 (PMC6954626; doi:10.1186/s13567-019-0731-2)
Supplement: Supplementary file 6 — Additional file 6. Distribution of PrP Sc in the CNS and peripheral tissues. [file 13567_2019_731_MOESM6_ESM.pdf]

Semi-quantitative scoring of PrP^{Sc} distribution in the brain

Genotype	Goat#	Olfactory lobe	Frontal cortex	N. caud/ putamen	Parietal cortex	Thalamus	Hippocampus	Midbrain	Cerebellum	Pons	Medulla oblongata	Trigeminal ganglion
PRNP$^{+/+}$	416	0.5	0.5	0.5	0.5	1.5	0.5	2.0	0.0	0.5	1.5	0.5
PRNP$^{+/+}$	417	2.5	1.0	1.0	1.5	3.0	3.0	3.0	2.5	3.0	2.5	1.0
PRNP$^{+/+}$	529	2.5	2.0	1.0	1.5	3.0	1.5	3.0	1.0	2.0	2.0	1.5
PRNP$^{+/+}$	536	1.0	0.5	0.5	0.5	0.5	0.5	2.0	0.5	1.5	1.5	0.5
		1.6	1.0	0.8	1.0	2.0	1.4	2.5	1.0	1.8	1.8	0.9
PRNP$^{+/Ter}$	451	3.0	2.5	2.0	2.5	2.5	2.5	2.0	0.5	1.0	0.5	NE
PRNP$^{+/Ter}$	469	NE	1.5	1.5	1.0	2.5	1.5	2.0	1.0	1.5	1.0	0.5
PRNP$^{+/Ter}$	527	3.0	2.5	1.5	2.0	2.5	2.0	2.0	1.5	2.5	2.5	1.0
PRNP$^{+/Ter}$	533	NE	2.0	1.5	2.0	2.5	1.5	2.0	0.5	2.0	1.5	1.0
		3.0	2.1	1.6	1.9	2.5	1.9	2.0	0.9	1.8	1.8	0.8
PRNP$^{Ter/Ter}$	413	0.0	0.0	0.0	0.0	0.0	0.0	0.0	0.0	0.0	0.0	0.0
PRNP$^{Ter/Ter}$	457	0.0	0.0	0.0	0.0	0.0	0.0	0.0	0.0	0.0	0.0	0.0
PRNP$^{Ter/Ter}$	476	0.0	0.0	0.0	0.0	0.0	0.0	0.0	0.0	0.0	0.0	0.0
PRNP$^{Ter/Ter}$	490	0.0	0.0	0.0	0.0	0.0	0.0	0.0	0.0	0.0	0.0	0.0
		0.0	0.0	0.0	0.0	0.0	0.0	0.0	0.0	0.0	0.0	0.0

Abbreviation: N. caud, Nucleus caudatus

Semi-quantitative scoring of PrP^{Sc} distribution in the spinal cord

		Cervical			Thoracal			Lumbal			Cauda equina		
**Genotype**	**Goat#**	DH	VH	DRG	DH	VH	DRG	DH	VH	DRG	DH	VH	DRG
PRNP$^{+/+}$	416	1.5	1.5	0.5	1.0	1.0	0.5	0.5	0.5	NE	1.0	0.5	0.0
PRNP$^{+/+}$	417	3.0	2.5	2.0	2.0	1.0	NE	3.0	2.0	2.0	2.5	0.5	1.0
PRNP$^{+/+}$	529	2.0	0.5	0.5	2.5	1.0	1.0	2.5	0.5	1.0	3.0	1.0	1.0
PRNP$^{+/+}$	536	1.5	1.0	NE	1.0	0.5	0.5	0.5	0.5	NE	1.5	0.5	NE
		2.0	1.4	1.0	1.6	0.9	0.7	1.6	0.9	1.5	2.0	0.6	0.7
PRNP$^{+/Ter}$	451	3.0	1.5	1.0	2.5	1.0	0.0	2.0	1.5	NE	2.5	2.0	0.5
PRNP$^{+/Ter}$	469	2.0	1.0	NE	1.5	0.5	0.5	2.0	1.5	0.5	NE	1.5	NE
PRNP$^{+/Ter}$	527	3.0	2.0	0.5	2.5	2.0	0.5	3.0	2.0	0.5	3.0	2.5	0.5
PRNP$^{+/Ter}$	533	1.5	1.0	0.0	1.0	1.0	1.0	1.5	1.5	0.0	3.0	2.5	1.0
		2.4	1.4	0.5	1.9	1.1	0.5	2.1	1.6	0.3	2.8	2.1	0.7
PRNP$^{Ter/Ter}$	413	0.0	0.0	0.0	0.0	0.0	0.0	0.0	0.0	0.0	0.0	0.0	0.0
PRNP$^{Ter/Ter}$	457	0.0	0.0	0.0	0.0	0.0	0.0	0.0	0.0	0.0	0.0	0.0	0.0
PRNP$^{Ter/Ter}$	476	0.0	0.0	0.0	0.0	0.0	0.0	0.0	0.0	0.0	0.0	0.0	0.0
PRNP$^{Ter/Ter}$	490	0.0	0.0	0.0	0.0	0.0	0.0	0.0	0.0	0.0	0.0	0.0	0.0
		0.0	0.0	0.0	0.0	0.0	0.0	0.0	0.0	0.0	0.0	0.0	0.0

Abbreviations: DH, dorsal horn; VH, ventral horn; DRG, dorsal root ganglion

Semi-quantitative scoring of PrP^{Sc} distribution in peripheral tissues

Genotype	Goat#	Parotid lymph node	RPLN	Superficial cervical lymph node	Spleen	RAMALT	Adrenal gland	Parotid gland
PRNP$^{+/+}$	416	NE	0.5	0.0	0.0	0.0	0.0	NE
PRNP$^{+/+}$	417	NE	0.5	0.0	0.0	0.0	0.0	NE
PRNP$^{+/+}$	529	NE	0.5	0.0	0.0	0.0	0.0	NE
PRNP$^{+/+}$	536	NE	2.0	0.0	0.0	0.0	0.0	NE
			0.9	0.0	0.0	0.0	0.0	
PRNP$^{+/Ter}$	451	0.0	0.0	0.0	0.0	0.0	0.0	0.0
PRNP$^{+/Ter}$	469	0.0	0.0	0.0	0.0	0.0	0.0	0.0
PRNP$^{+/Ter}$	527	1.0	0.0	0.0	0.0	0.0	0.0	0.0
PRNP$^{+/Ter}$	533	NE	0.0	NE	0.0	0.0	0.0	0.0
		0.3	0.0	0.0	0.0	0.0	0.0	0.0
PRNP$^{Ter/Ter}$	413	0.0	0.0	0.0	0.0	0.0	0.0	0.0
PRNP$^{Ter/Ter}$	457	0.0	0.0	0.0	0.0	0.0	0.0	0.0
PRNP$^{Ter/Ter}$	476	0.0	0.0	0.0	0.0	0.0	0.0	0.0
PRNP$^{Ter/Ter}$	490	0.0	0.0	0.0	0.0	0.0	0.0	0.0
		0.0	0.0	0.0	0.0	0.0	0.0	0.0

Abbreviations: RPLN, retropharyngeal lymph node; RAMALT, rectoanal mucosa-associated lymphoid tissue
